# Possible Role of Dorsolateral Prefrontal Cortex in Error Awareness: Single-Pulse TMS Evidence

**DOI:** 10.3389/fnins.2018.00179

**Published:** 2018-03-21

**Authors:** Fabio Masina, Antonino Vallesi, Elisa Di Rosa, Luca Semenzato, Daniela Mapelli

**Affiliations:** ^1^Department of General Psychology, University of Padova, Padova, Italy; ^2^Department of Neuroscience, Padua Neuroscience Center, University of Padova, Padova, Italy; ^3^San Camillo Hospital IRCCS, Venice, Italy; ^4^Department of Neuroscience, University of Padova, Padova, Italy; ^5^Human Inspired Technologies Research Center, University of Padova, Padova, Italy

**Keywords:** error awareness, transcranial magnetic stimulation, dorsolateral prefrontal cortex, on-line TMS, null findings

## Abstract

**Background:** Error awareness is essential to maintain an adaptive and goal-directed behavior and is supposed to rely on the activity of the right dorsolateral prefrontal cortex (DLPFC). However, studies employing electrophysiological methods and functional resonance imaging (fMRI) do not allow to establish a causal relationship between error awareness and implicated brain structures.

**Objective:** The study examined the causal relationship between DLPFC activity and error awareness in order to confirm the involvement of the right DLPFC in error awareness and to obtain temporal information about this process, namely when the activity of the right DLPFC is involved in error awareness.

**Methods:** Three experiments with three different samples were conducted employing on-line Transcranial Magnetic Stimulation (TMS). A paired-pulse and a single-pulse on-line TMS paradigm were employed respectively in Experiments 1 and 3, whereas in Experiment 2 a control test was conducted without TMS. In TMS experiments, the right DLPFC was stimulated, considering the left DLPFC and the Vertex as control sites.

**Results:** Experiment 1 showed no effect of paired-pulse TMS over either right or left DLPFC on error awareness. In Experiment 3, independently from the time point during which TMS was delivered, results showed a significant effect of single-pulse TMS over the DLPFC on Stroop Awareness, without evidence for lateralization of the process.

**Conclusions:** Results of the present study partially demonstrate the involvement of the DLPFC in error awareness.

## Introduction

“*Among errors, there are those that stink of sewage and the ones that smell of laundry,”* wrote Indro Montanelli, a famous journalist and author (Montanelli, 1992). This sentence efficaciously expresses both the salience and the functional role of certain errors in daily life. Actually, although the detection of an error can be annoying because it forces us to adjust our behavior, it is crucial to maintain an adaptive and goal-directed behavior, and to avoid erroneous and potentially detrimental actions. However, contrary to common sense, we are not aware of all errors we make.

A terminological distinction between error monitoring and error awareness derives from the literature. On the one hand, in the literature, the term “error monitoring” refers to a multi-componential system that contributes to refocus attention to the task, triggering behavioral adjustments and emotional reaction following an error (Taylor et al., 2007), regardless of whether the error made was aware or not. On the other hand, the term “error awareness” indicates more specifically a process that allows the conscious detection of an error (Ullsperger et al., 2014). Therefore, according to the aim of a study, these two terms can be used to indicate a cognitive system that processes the error information (i.e., error monitoring) or a metacognitive system that allows being aware of an error (i.e., error awareness).

Several studies from cognitive neuroscience have demonstrated that error monitoring is not a synonym of error awareness (Endrass et al., 2005). First of all, studies employing event-related potentials (ERPs), have revealed that the error information is processed in at least two phases: a first one, dedicated to a rough processing of an error, and a second one, more related to a proper evaluation and conscious perception of the error (Wessel, 2012). According to this distinction, these studies have shown a difference between aware and unaware errors in the ERP components. In fact, while the Error Related Negativity, i.e., a fronto-central deflection recorded between 20 and 100 ms after an erroneous response, is more related to the first phase, the Error Positivity, a more posterior positive deflection that peaks 100–200 ms after an erroneous response, is generally larger when the error is consciously perceived with respect to when it is not (Nieuwenhuis et al., 2001; Endrass et al., 2007).

Complementary to this evidence, recent neuropsychological studies and studies employing both structural and functional resonance imaging (MRI) have tried to understand more in detail the neural substrate of error awareness, showing that this capacity is correlated with the activity of several cortical and sub-cortical structures such as the anterior cingulate cortex (van Veen and Carter, 2002; Hester et al., 2009; Maier et al., 2015), the thalamus (Seifert et al., 2011), the anterior insula (Klein et al., 2007, 2013), and the prefrontal cortex (Hoerold et al., 2013).

In addition, a number of electrophysiological (EEG) studies using source analyses have identified cortical structures correlated with error awareness. However, evidence from these studies is not always convergent. For example Charles et al. (2013) have revealed a relationship between aware errors and the activity of the posterior cingulate cortex, as well as a correlation between unaware errors and the dorsal anterior cingulate cortex, whereas O'Connell et al. (2007) have pointed out the role of the anterior cingulate cortex in both aware and unaware error detection.

Taken together, these studies seem to depict error awareness as a complex process, which would emerge only after a first rough processing of the error information, and that would be related to the activity of a broadly distributed brain network. However, results obtained with ERP, neuroimaging, and EEG methods, despite their good spatial or temporal resolution, do not allow to establish a causal relationship between structure and function, because they provide only correlational evidence. Moreover, the results of the neuropsychological investigations carried out so far (e.g., Hoerold et al., 2013) lack a fine-grained specificity due to the broad extent of the reported lesions.

To overcome these methodological limitations, non-invasive brain stimulation (NIBS) has recently been employed, obtaining promising results. In detail, using transcranial Direct Current Stimulation (tDCS), Harty et al. (2014) have suggested the presence of a causal relationship between error awareness and the activity of the right dorsolateral prefrontal cortex (DLPFC), a brain area that seems to be implicated in awareness of cognitive functioning (Fleming et al., 2012). The authors tested a group of healthy older adults (65–86 years), who presented a low error awareness (they were aware of about 50% of their errors) and ran 4 separate experiments in order to test the effect of both anodal and cathodal stimulation over the right and left DLPFC. What the authors showed in Experiment 1, and replicated in Experiment 4, is that the application of anodal tDCS over the right DLPFC was associated with a significant enhancement of error awareness in elderly performing the Error Awareness Task (EAT, Hester et al., 2005). In the literature about the neural bases of error awareness, these results represent the first evidence of a causal relationship between structure (right DLPFC) and function (error awareness).

However, two important limitations of that study (Harty et al., 2014) need to be acknowledged. Specifically, the first limitation concerns the low spatial resolution of these results. In fact, according to a recent study (Cieslik et al., 2013), the DLPFC can be divided into at least two subregions: an anterior subregion, more associated with attention and cognitive control, therefore more related to error awareness, and a posterior subregion, more associated with working memory. Considering this complexity of the DLPFC, the methodology adopted by Harty et al. (2014) does not allow establishing which part of the right DLPFC was really involved in error awareness. Aside this consideration, the study of Cieslik et al. (2013) is particularly interesting because it could partially explain results provided by Harty et al. (2014). In fact, as Cieslik et al. (2013) showed, the anterior subregion of the right DLPFC seems to be functionally connected with the ACC, another brain area strongly correlated to error awareness (van Veen and Carter, 2002; Hester et al., 2009; Maier et al., 2015). Therefore, it is reasonable to hypothesize that the stimulation of the right DLPFC could indirectly have modulated the ACC.

A second potential limitation of Harty's results, again due to the technique employed, concerns the absence of any temporal information about error awareness, specifically when the activity of the right DLPFC was involved in error awareness.

With the aim to overcome these two important limitations, in the present study both a different NIBS technique and a neuronavigation system were employed. Specifically, in order to investigate temporal information about error awareness, we employed the Transcranial Magnetic Stimulation (TMS), opting for an on-line TMS paradigm. Unlike tDCS, which relies on an accumulation of ionic gradients requiring several minutes to produce detectable effects, in on-line single-pulse TMS paradigms, pulses are discrete events that produce a punctual neuronal depolarization (Wagner et al., 2007) and can, therefore, be used to infer the timing of neural and cognitive events. Furthermore, because the identification of targets by a neuronavigation system allows a better location with respect to the 10-20 international system (Carducci and Brusco, 2012), we localized both the right and left DLPFC by two methods: (1) the individual magnetic resonance images (MRIs); (2) when the acquisition of MRIs for a particular individual was precluded, a magnetic resonance-based head model constructed by spatially deforming a standard averaged magnetic resonance template. In the present study, the right and left DLPFC were identified through the spatial coordinates proposed by Cieslik et al. (2013).

The purpose of the present study was to confirm the involvement of the right DLPFC in error awareness, as well as to shed light on the timing of error awareness. In this study, three experiments were conducted: a paired-pulse and a single-pulse on-line stimulation paradigms were employed respectively in Experiments 1 and 3, whereas a control test was conducted without stimulation (Experiment 2). All three experiments were conducted according to the declaration of Helsinki and were approved by the Ethics Committee of the School of Psychology, University of Padua. All the participants enrolled in experiments were volunteers and did not receive any reimbursement. Before experiment, participants gave their written informed consent and were checked for TMS exclusion criteria (Rossi et al., 2011). The adopted safety procedures were in line with the guidelines for the use of TMS (Rossi et al., 2009).

## Experiment 1

### Methods

#### Participants

Twenty volunteers participated in Experiment 1 (6 male, 22.5 ± 3.2 years, range: 19–30). All participants had a normal or corrected-to-normal vision and were right-handed. Each participant took part in three experimental sessions carried out on different days (3 days on average were left between each session). During each session, only a brain site was stimulated (e.g., Session 1: right DLPFC, Session 2: left DLPFC; Session 3: Vertex).

#### Error awareness measures

**Figure 1:**
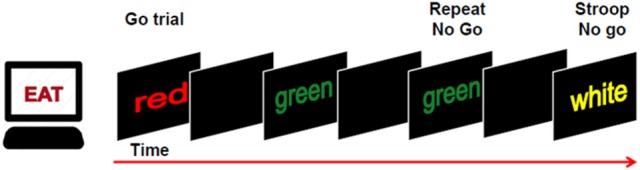
Participants were required to make a speeded response (“button 3”) to all congruent trials (word and color) and to withhold the response to incongruent trials or when a word was repeated. Participants were also required to signal an error by pressing a different button (“space bar”).

In order to evaluate error awareness, a modified version of the EAT was adopted. The EAT is a motor Go/No-go response inhibition task in which a serial stream of single color words in colored fonts are presented (Figure 1). Participants were trained to respond with a single-speeded press of a button (“3” on the keyboard), with the left hand, when the semantic meaning of the word and its font color were congruent (Go trial), while they were asked to withhold the response in two circumstances: (1) when the semantic meaning of the word and its font color were incongruent (Stroop No-go trial); (2) when the word presented on the current trial was the same as the one presented previously (Repeat No-go trial). Moreover, participants were instructed to signal an error commission, both Stroop and Repeat errors, by pressing the space bar with the right hand. According with previous studies (O'Connell et al., 2009; Murphy et al., 2012; Harty et al., 2014), participants could signal an error immediately after its commission, instead of delaying this response for a fixed time as it was the case for other studies in which the EAT was used (Shalgi et al., 2007; Hester et al., 2009; Harty et al., 2013). This expedient allowed measuring the timing of error awareness (*error awareness RT*) as well as the error awareness itself. In addition, similarly to a prior study (Harty et al., 2013), we used an adaptive staircase approach to maintain the number of errors between subjects as similar as possible. To this aim, in the present study, the task difficulty was based on the participants' accuracy on No-go trials. At the beginning of the task, the word was presented for 750 ms with an interstimulus interval (ISI) of 1,250 ms. These durations were maintained if the accuracy was between 50 and 60%. When the accuracy on No-go fell under 50%, the presentation of the word and ISI were both set to 1,000 ms, whereas when the accuracy on No-go exceeded 60%, the presentation of the word and ISI were respectively set to 500 and 1,500 ms. During the task, this check of accuracy was computed after each No-go trial. Stimuli appeared at the center of the screen on a black background. The total number of trials in the task was 1,150, specifically 1,000 Go trials, 75 Repeat No-go trials, and 75 Stroop No-go trials. The task was divided into five blocks including 230 trials each. It was ensured that all participants were well-trained and fully understood the instructions of the task before they began experiment. Participants rested their head on a table-mounted head-rest which fixed their distance at 60 cm from a 19-inch monitor for the duration of the task. The response device was a PS2 standard keyboard. Stimulus presentation was controlled by E-Prime software (Psychological Software Tools, Pittsburgh, PA, USA; version 2.0.8.90).

#### TMS

TMS pulses were delivered via a Magstim Rapid^2^ TMS stimulator (Magstim Company, Whitland, UK). A 70-mm figure-of-eight stimulation coil was fixed in space thanks to well-trained operators over target brain sites. In both the right and left DLPFC sessions the coil was oriented with the handle at 45° to the mid-sagittal line. In the Vertex session, the coil was positioned with the handle pointing backwards parallel with the midline. Since TMS over the frontal sites could be annoying, the intensity of magnetic stimulation was prudentially set 5% below the individual motor threshold. The intensity was estimated by the observed movement motor threshold (OM-MT) method (Pridmore et al., 1998). The stimulation targets were identified with Brainsight frameless stereotaxic system (Rogue Research, Montreal, Canada) and spatial transformation was used to adjust the MRI template (the non-linear ICBM-152 template by the Montreal Neurological Institute) to individual head shapes. According to Cieslik et al. (2013), the coordinates of the right and left DLPFC were ±30, 43, 23 (MNI coordinates). Since one of the aims of the study was to confirm the involvement of the right DLPFC in error awareness, the left DLPFC was a control area. For this reason, we decided to target the left DLPFC by using the same coordinates we adopted to identify the right DLPFC (MNI coordinates: 30, 43, 23), but changing only the x-parameter (MNI coordinates: −30, 43, 23), a strategy to select a control site that has already been used in previous TMS studies (Herwig et al., 2003; Vallesi et al., 2007). The Vertex corresponded to the Cz site of the international 10–20 system (Steinmetz et al., 1989). The order of the stimulation sites was randomly assigned to each participant, for example, PARTICIPANT_1 (Session 1: right DLPFC, Session 2: left DLPFC; Session 3: Vertex), PARTICIPANT_2 (Session 1: left DLPFC, Session 2: right DLPFC; Session 3: Vertex), and so on. In total, in Experiment 1, we collected data from 60 TMS sessions (20 participants × 3 sessions).

**Figure 2:**
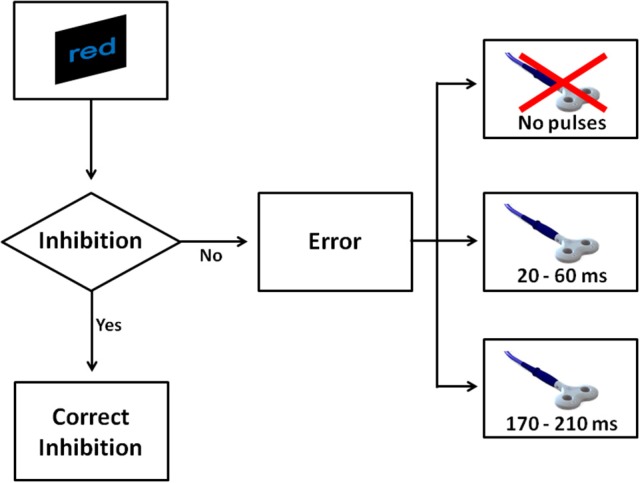
The figure shows all possible scenarios when a No-go trial appeared in Experiment 1. After a No-go, four scenarios were possible. If the participant withheld the response, this was considered a correct inhibition. In the case the participant made a mistake, one of three events could occur: a couple of TMS pulses was delivered at 20 and 60 ms, alternatively a couple of pulses was delivered at 170 and 210 ms, or alternatively no TMS pulse was delivered.

During the task, pairs of TMS pulses (with 40 ms between the two pulses) were delivered as an attempt to produce greater effects than single-pulse TMS, as previous studies reported (O'Shea et al., 2004; Bardi et al., 2012). TMS pulses were delivered in two possible time windows after an error commission: 20–60 ms or 170–210 ms (Figure 2). Furthermore, pairs of TMS pulses were also delivered at 110–150 ms after a correct response. Notably, TMS pulses were always triggered by a response, but not all the responses triggered TMS pulses. In fact, the delivery of TMS pulses was predetermined. Specifically, only 80% of No-go trials and 40% of Go trials triggered TMS pulses.

#### Data analysis

##### Mean error awareness

The *mean error awareness* was computed dividing the number of aware errors by the total number of errors (O'Connell et al., 2009). Error awareness for Stroop and Repeat errors was computed separately since previous studies using the EAT have found higher error awareness for Stroop compared with Repeat errors (O'Connell et al., 2007; Hester et al., 2009; Harty et al., 2014). Therefore, we included in a repeated-measures 2 × 3 × 3 ANOVA “trial type” (Stroop vs. Repeat), “timing of TMS pulses” (no pulse, 20–60 ms, and 170–210 ms), and “site of stimulation” (right DLPFC, left DLPFC, and Vertex) as within-subject factors. In this analysis, the sample size was reduced to 18 participants because 2 of them did not commit any Repeat errors within some conditions of the analysis. Since the reduction of the sample size can increase the Type 2 error rate, we decided to conduct two different repeated-measures 3 × 3 ANOVAs, in order to evaluate separately the effect of TMS on Stroop and Repeat errors.

The first 3 × 3 ANOVA (sample size: *n* = 20) considered only the Stroop errors with “timing of TMS pulses” (no pulse, 20–60 ms, and 170–210 ms) and “site of stimulation” (right DLPFC, left DLPFC, and Vertex) as within-subject factors.

The second 3 × 3 ANOVA (sample size: *n* = 18) took only the Repeat errors into consideration, again with “timing of TMS pulses” (no pulse, 20–60 ms, and 170–210 ms) and “site of stimulation” (right DLPFC, left DLPFC, and Vertex) as within-subject factors.

##### Error awareness RTs

*Error awareness RTs* were computed as the time between the error commission and its detection (when the participant pressed the space bar to signal an error). A repeated-measures 3 × 3 ANOVA was conducted with “timing of TMS pulses” (no pulse, 20–60 ms, and 170–210 ms) and “site of stimulation” (right DLPFC, left DLPFC, and Vertex) as within-subject factors.

##### Mean RTs

*Mean RTs* related to correct responses and errors were analyzed in a repeated-measures 2 × 3 ANOVA considering “kind of response” (Go RTs vs. error RTs) and “site of stimulation” (right DLPFC, left DLPFC, and Vertex) as within-subject factors.

##### Mean accuracy

Finally, *mean accuracy* was calculated as the ratio of correct withholds on No-go trials. The *mean accuracy* was analyzed by a repeated-measures ANOVA with “site of stimulation” (right DLPFC, left DLPFC, and Vertex) as within-subject factor.

Before data analyses, all RTs above and below 2 standard deviation (SD) from the mean were excluded and a logarithm transformation was applied on the remaining RTs, in order to improve normalization. In every analysis, the Bonferroni correction for multiple comparisons was applied and a corrected alpha-level of 0.05 was considered. Finally, effect sizes were estimated by partial eta squared (η^2^_*p*_).

### Results

The behavioral measures of Experiment 1 are presented in Table 1.

**Table 1:** Mean and standard deviation (*SD*) of performance indices on the EAT for right DLPFC, left DLPFC, and Vertex stimulation.

	**Right DLPFC**	**Left DLPFC**	**Vertex**
	**Mean (*****SD*****)**	**Mean (*****SD*****)**	**Mean (*****SD*****)**
Stroop Awareness (%)	94 (10)	95 (10)	94 (10)
Repeat Awareness (%)	83 (10)	82 (20)	81 (20)
Error Awareness RT (ms)	407 (95)	408 (94)	407 (92)
Go RT (ms)	471 (66)	482 (56)	479 (70)
Error RT (ms)	448 (56)	453 (55)	449 (62)
Accuracy (%)	51 (20)	52 (20)	52 (20)

*DLPFC, Dorsolateral prefrontal cortex*.

#### Mean error awareness

The number of errors in each condition is shown in Table 2.

**Table 2:** Mean and standard deviation (*SD*) of the number of errors in each condition.

**Condition**	**Right DLPFC**	**Left DLPFC**	**Vertex**
	**Mean (*****SD*****)**	**Mean (*****SD*****)**	**Mean (*****SD*****)**
Errors	72 (30)	71 (25)	70 (30)
Stroop Errors	44 (17)	43 (12)	43 (16)
Stroop Errors_no pulse	7 (3)	7 (2)	7 (3)
Stroop Errors_20–60 ms_TMS	18 (7)	18 (5)	19 (7)
Stroop Errors_170–210 ms_TMS	18 (7)	18 (5)	17 (7)
Repeat Errors	29 (16)	28 (15)	27 (16)
Repeat Errors_no pulse	6 (3)	5 (3)	4 (3)
Repeat Errors_20–60 ms_TMS	11 (7)	11 (6)	11 (7)
Repeat Errors_170–210 ms_TMS	12 (7)	12 (6)	12 (7)

*DLPFC, Dorsolateral prefrontal cortex*.

Results from the first 2 × 3 × 3 ANOVA showed a main effect of “trial type” [*F*_(1, 17)_ = 13.8, *p* < 0.01, $\eta_{\text{p}}^{2}$ = 0.4]. As expected, participants were more aware for Stroop than Repeat errors. No other main effect or interaction reached statistical significance (lowest *p*-value = 0.1).

In order to evaluate separately the effect of TMS on Stroop and Repeat errors, two separate repeated-measures 3 × 3 ANOVAs were conducted. Both these 3 × 3 ANOVAs did not reveal main effects or interactions (lowest *p*-value = 0.4).

#### Error awareness RTs

The analysis did not show any effect of TMS on *error awareness RTs* (lowest *p*-value = 0.3).

#### Mean RTs

The analysis of *mean RTs* yielded a significant difference between Go RTs and error RTs [*F*_(1, 19)_ = 73.7, *p* < 0.001, $\eta_{\text{p}}^{2}$ = 0.8], indicating that error RTs were faster than Go RTs. No main effect or interaction with the factor “site of stimulation” was found (lowest *p*-value = 0.3).

#### Mean accuracy

Finally, no significant effect of TMS was found on *mean accuracy* (lowest *p*-value = 0.8).

### Discussion

Results of Experiment 1 showed no significant effect of paired-pulse TMS over either left or right DLPFC on error awareness. Several explanations could have produced these null results and a quite obvious reason could depend on the inadequacy of the target area. However, Harty et al. (2014) had strongly demonstrated in two experiments the involvement of the right DLPFC in error awareness.

Another possible explanation could be the employment of an inadequate stimulation paradigm. With regard to this last point, a problem related to TMS concerns all kinds of non-specific effects that TMS can produce. Even if TMS is a relatively painless method, it generates somatosensory sensations that can nonspecifically alter task performance (Robertson et al., 2003), producing artifacts independently from the brain site stimulated in a specific circumstance. During on-line TMS paradigm, participants can, in fact, shift their attention from the task to the TMS pulse. Considering in these terms the TMS paradigm we used in Experiment 1, it is plausible to suppose that an annoying paired-pulse delivered during the execution of a task could have increased the arousal of participants. This hypothesis is not new in literature because, among others, also Dräger et al. (2004) revealed this non-specific effect due to TMS.

Taken together these considerations, we believed that a fruitful strategy to disambiguate confounding non-specific effects of TMS from specific effects induced by this technique was to compare Experiment 1 with a second experiment in which TMS was not delivered.

## Experiment 2

### Methods

#### Participants

In Experiment 2, 20 healthy participants were recruited. All participants were right-handed and had normal or correct-to-normal vision. Because of an unusual *mean error awareness* (< 30%), a participant was excluded from the analyses. As a result, the final sample consisted of 19 participants (5 men, 23.8 ± 3.3 years, range: 19–29). Each participant performed the EAT and received the same instructions as in Experiment 1. All participants were tested in one experimental session without TMS.

#### Data analysis

The behavioral measures collected from this control group were compared with the ones of Experiment 1. Since participants in Experiment 1 performed the EAT three times (once for each session), in order to avoid practice effect, we compared the behavioral measures of Experiment 2 with the measures at the first session collected in Experiment 1. For the sake of clarity, in Experiment 1, sites of stimulation in the first session were so distributed: right DLPFC (*n* = 7), left DLPFC (*n* = 7), Vertex (*n* = 6).

##### Mean error awareness, error awareness RTs, mean RTs and mean accuracy

Mean error awareness, error awareness RTs, mean RTs and mean accuracy from both experiments were compared by one-way ANOVAs with “group” (Experiment 1 vs. Experiment 2) as between-subject factor. As in Experiment 1, RTs above and below 2 standard deviation (SD) were not included in the analyses. Moreover, a logarithm transformation was used on the remaining RTs, to increase normalization. A corrected alpha-level of 0.05 was considered in each analysis and the effect sizes were estimated by partial eta squared (η^2^_*p*_).

### Results

The behavioral measures of both studies are shown in Table 3.

**Table 3:** Mean and standard deviation (*SD*) of performance indices on the EAT for the first and second experiment.

	**Experiment 1 - TMS (first session)**	**Experiment 2 - no TMS**	
	**Mean (*****SD*****)**	**Mean (*****SD*****)**	***F*****-values**
Stroop Awareness (%)	93 (10)	91 (10)	0.6
Repeat Awareness (%)	82 (20)	79 (20)	0.3
**Error Awareness RT (ms)**	**445 (101)**	**503 (92)**	**4.3**
Go RT (ms)	515 (50)	496 (115)	1.2
Error RT (ms)	462 (50)	451 (116)	0.7
Accuracy (%)	53 (20)	58 (20)	0.5

*In bold, statistically significant differences between groups (p < 0.05)*.

The statistical analyses revealed that *error awareness RTs* were different between groups, [*F*_(1, 38)_ = 4.3, *p* < 0.05, η^2^_*p*_ = 0.1]. Participants in Experiment 1 were faster to signal their errors than participants in Experiment 2. The other one-way ANOVAs did not reveal differences between groups (lowest *p*-value = 0.3).

### Discussion

In Experiment 2, participants performed the EAT in absence of TMS. Data from this experiment were then compared to data from first sessions of Experiment 1 (TMS experiment).

Results showed that the effect of TMS in Experiment 1, independently from the site of stimulation, produced a reduction of the time needed to signal an error (*error awareness RTs*). This non-specific TMS-induced effect on RTs is not unusual, in fact, a speeding effect associated with TMS has been also reported in previous studies (Terao et al., 1997; van Campen et al., 2013). Since every TMS pulse is linked to a clearly noticeable sensation on the head and a clicking sound, it is reasonable to consider that these somatosensory sensations could also influence task performance and potentially increase the arousal, as Dräger et al. (2004) revealed. This previous evidence might support an explanation for null findings we found in Experiment 1. In fact, in Experiment 1, participants received, after a response, pairs of TMS pulses and, independently from the sites on which TMS was delivered, the paradigm of stimulation may have increased the level of arousal, submerging any specific effect of TMS on error awareness. Although in Experiment 1 we expected that the paradigm of stimulation would have maximized the behavioral effects of TMS on error awareness (O'Shea et al., 2004; Bardi et al., 2012), the high number of pulses delivered during the task might have been the reason of the increase of the arousal, that in turn would have submerged specific effects of TMS. This consideration is particularly important if considering the fact that error awareness is a cognitive process particularly sensitive to arousal (Shalgi et al., 2007; Robertson, 2014).

Although suggestive, these interpretations still cannot provide an answer to a crucial question: did null results in Experiment 1 depend on the inefficacy of the site of stimulation? To answer to this question, a third experiment was implemented, setting a paradigm of stimulation characterized by fewer pulses than the paradigm in Experiment 1, in order to minimize the hypothesized impact of the arousal on error awareness.

## Experiment 3

### Methods

#### Participants

Twenty right-handed healthy individuals, with a normal or corrected-to-normal vision, participated in Experiment 3 (5 men, 24.6 ± 2.9 years, range: 21–31). Participants were involved in 3 sessions and 3 days on average were left between each session. During each session, only a brain site was stimulated (e.g., Session 1: right DLPFC, Session 2: left DLPFC; Session 3: Vertex). All participants performed the EAT. Structure and instructions of the task were the same as Experiment 1 and 2.

#### TMS

TMS stimulator, type of coil, placing of the coil, and method to measure the individual motor threshold were identical to those of Experiment 1. In Experiment 3, the TMS intensity was set at 100% of the individual motor threshold, to increase a possible effect of TMS and also in the light that here the TMS protocol was overall less intensive than Experiment 1 (single pulse vs. paired pulse). In total, in Experiment 3, we collected data from 60 TMS sessions (20 participants × 3 sessions). The MNI coordinates of the right and left DLPFC were identical to Experiment 1, namely ±30, 43, 23 (MNI coordinates), as well as the position of the control site (Vertex). In addition, in Experiment 3, the cortical location of sites was visually verified by Brainsight frameless stereotaxy (Rogue Research, Montreal, QC, Canada) on T1-weighted MRIs of 11 participants. Images were acquired using a 3-T Philips Ingenia whole-body scanner with a 32-channel head-coil at the Neuroradiology Unit, University-Hospital of Padova, Italy. MRIs were then registered to the MNI template. For the extra 9 participants, the localization of sites was based on individualized MRI template by a magnetic resonance-based head model, as all participants in Experiment 1.

**Figure 3:**
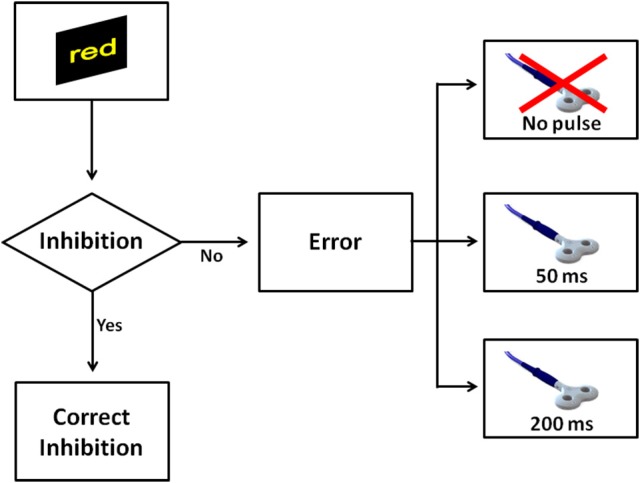
The figure shows all possible scenarios when a No-go trial appeared in Experiment 3. Similarly to Experiment 1, a correct inhibition was considered when a participant withheld the response on No-go. In the case the participant made a mistake, one of three events could occur: a single pulse was delivered at 50 ms, a single pulse was delivered at 200 ms, or finally no TMS pulse was delivered.

In Experiment 3, we maintained same procedures of Experiment 1, apart from the paradigm of stimulation. To minimize a possible confound of non-specific effects of TMS and to reduce the impact of a supposed increase of the arousal level induced by TMS, in Experiment 3 we adopted a single-pulse TMS paradigm, instead of a paired-pulse. Thus, this paradigm of stimulation was characterized by an overall reduced number of TMS pulses per session (50%) than the paradigm adopted in Experiment 1. A single-pulse TMS was delivered in two time windows after an error commission: 50 or 200 ms (Figure 3). Furthermore, a single-pulse TMS was also delivered at 125 ms after a correct response. The probability to receive a TMS pulse after an error was again 80 and 40% after a Go trial.

#### Data analysis

##### Mean error awareness.

The analyses on *mean error awareness* were the same as Experiment 1. A repeated-measures 2 × 3 × 3 ANOVA was conducted, with “trial type” (Stroop vs. Repeat), “timing of TMS pulses” (no pulse, 50 ms, and 200 ms), and “site of stimulation” (right DLPFC, left DLPFC, and Vertex) as within-subject factors. As in Experiment 1, this analysis yielded to a reduction of the sample size (*n* = 15) because 5 participant did not commit any Repeat commission errors within some conditions of the analysis. Therefore, exactly for the same reasons of Experiment 1, namely to avoid an increase of the Type 2 error rate, we evaluated separately the effect of TMS on Stroop and Repeat commission errors by means of two repeated-measures 3 × 3 ANOVAs.

The first 3 × 3 ANOVA (sample size: *n* = 20) considered only the Stroop commission errors with “timing of TMS pulses” (no pulse, 50 ms, and 200 ms) and “site of stimulation” (right DLPFC, left DLPFC, and Vertex) as within-subject factors. Since this analysis showed a borderline significant interaction, we decided to reduce the model and collapse the factor “timing of TMS pulses” (no pulse, 50 ms, and 200 ms) in a dichotomous factor “TMS” (no pulse vs. TMS pulse). Finally, the second 3 × 3 ANOVA (sample size: *n* = 15) included only the Repeat commission errors, with “timing of TMS pulses” (no pulse, 50 ms, and 200 ms) and “site of stimulation” (right DLPFC, left DLPFC, and Vertex) as within-subject factors.

##### Error awareness RTs

Error awareness RTs were analyzed by a repeated-measures 3 × 3 ANOVA with “timing of TMS pulses” (no pulse, 50 ms, and 200 ms) and “site of stimulation” (right DLPFC, left DLPFC, and Vertex) as within-subject factors.

##### Mean RTs

Mean RTs were analyzed in a repeated-measures 2 × 3 ANOVA considering “kind of response” (Go RTs vs. error RTs) and “site of stimulation” (right DLPFC, left DLPFC, and Vertex) as within-subject factors.

##### Mean accuracy

Finally, *mean accuracy* was analyzed by a repeated-measures ANOVA with “site of stimulation” (right DLPFC, left DLPFC, and Vertex) as within-subject factor.

Reaction times above and below 2 standard deviation (SD) were excluded from the analyses and a logarithm transformation was applied on the remaining RTs. The Bonferroni correction was applied to post hoc analyses. Effect sizes were calculated in terms of partial eta squares (η^2^_*p*_).

### Results

The behavioral measures of Experiment 3 are summarized in Table 4.

**Table 4:** Mean and standard deviation (*SD*) of performance indices on the EAT for right DLPFC, left DLPFC, and Vertex stimulation.

	**Right DLPFC**	**Left DLPFC**	**Vertex**
	**Mean (*****SD*****)**	**Mean (*****SD*****)**	**Mean (*****SD*****)**
Stroop Awareness (%)	96 (0)	95 (10)	97 (10)
Repeat Awareness (%)	79 (10)	79 (20)	81 (10)
Error Awareness RT (ms)	384 (82)	398 (89)	415 (76)
Go RT (ms)	462 (61)	467 (56)	458 (39)
Error RT (ms)	436 (46)	442 (42)	435 (36)
Accuracy (%)	58 (10)	55 (10)	58 (10)

*DLPFC, Dorsolateral prefrontal cortex*.

#### Mean error awareness

The number of errors in each condition is presented in Table 5.

**Table 5:** Mean and standard deviation (*SD*) of the number of errors in each condition.

**Condition**	**Right DLPFC**	**Left DLPFC**	**Vertex**
	**Mean (*****SD*****)**	**Mean (*****SD*****)**	**Mean (*****SD*****)**
Errors	59 (20)	63 (18)	59 (20)
Stroop Errors	38 (11)	41 (10)	38 (11)
Stroop Errors_no pulse	5 (2)	5 (2)	5 (1)
Stroop Errors_50 ms_TMS	15 (4)	17 (3)	16 (4)
Stroop Errors_200 ms_TMS	17 (6)	19 (5)	16 (6)
Repeat Errors	21 (11)	21 (11)	21 (11)
Repeat Errors_no pulse	4 (2)	4 (3)	5 (3)
Repeat Errors_50 ms_TMS	9 (5)	8 (5)	7 (5)
Repeat Errors_200 ms_TMS	8 (5)	10 (5)	9 (5)

*DLPFC, Dorsolateral prefrontal cortex*.

The first repeated-measures 2 × 3 × 3 ANOVA revealed a main effect of “trial type” [*F*_(1, 14)_ = 37.4, *p* < 0.001, η^2^_*p*_ = 0.7]. As in Experiment 1, participants were more aware for Stroop than Repeat errors. No other main effect or interaction was found (lowest *p*-value = 0.3).

**Figure 4:**
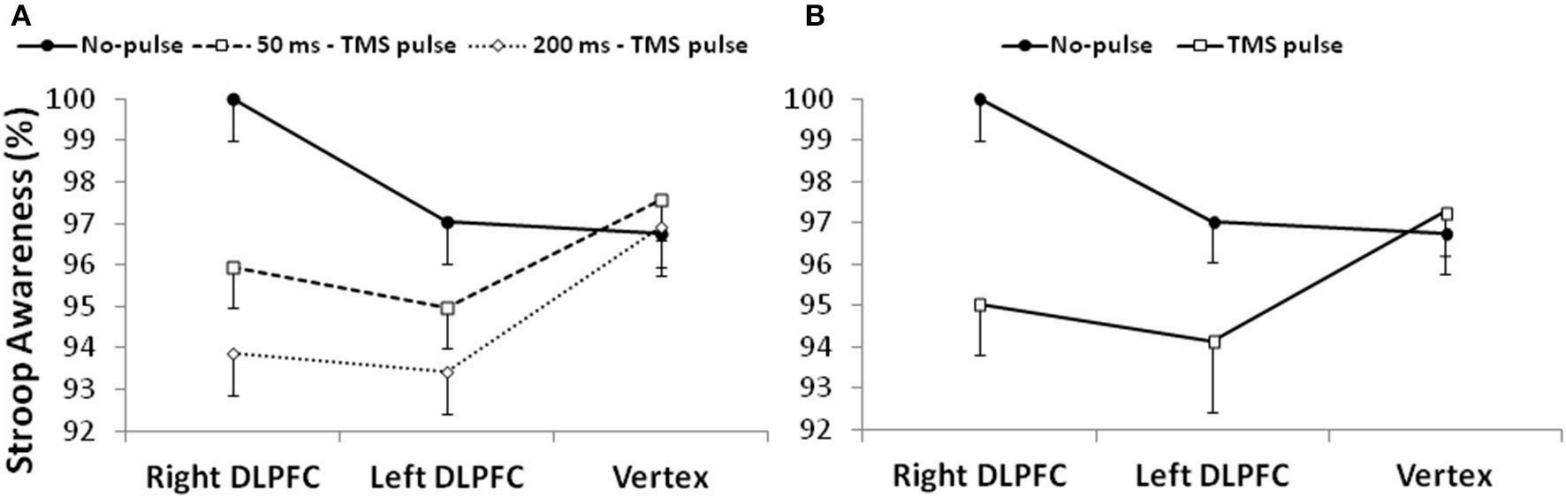
**(A)** The left side of the figure shows Stroop Awareness (%) after three possible conditions: no TMS pulse, a pulse delivered at 50 ms, and a pulse delivered at 200 ms; **(B)** the right side of the figure shows Stroop Awareness (%) after collapsing the factor “timing of TMS pulses.” DLPFC, Dorsolateral prefrontal cortex.

When two different repeated-measures 3 × 3 ANOVAs were conducted on Stroop and Repeat errors, the first 3 × 3 ANOVA on Stroop errors shown a main effect of “timing of TMS pulses” [*F*_(2, 38)_ = 8.1, *p* < 0.01, $\eta_{\text{p}}^{2}$ = 0.3]. The corrected paired sample comparisons indicated that participants were more aware when they did not receive any TMS pulse after an error commission (no pulse condition) than the condition in which the pulse was delivered at 200 ms after an error commission [respectively 98 vs. 95%; *t*_(19)_ = 4, *p* < 0.01; Figure 4A]. No other main effect or interaction was found (lowest *p*-value = 0.1). However, after collapsing the factor “timing of TMS pulses” (no pulse, 50 ms, and 200 ms) in a dichotomous factor “TMS” (no pulse vs. TMS pulse), the analysis for Stroop Awareness showed a significant interaction between “TMS” x “site of stimulation” [*F*_(2, 38)_ = 3.61, *p* < 0.05, $\eta_{\text{p}}^{2}$ = 0.2]. Corrected paired sample *t*-tests indicated that the interaction was driven by a reduction of Stroop Awareness in both the right and left DLPFC stimulation sessions [right DLPFC: *t*_(19)_ = 4.2, *p* < 0.01; left DLPFC: *t*_(19)_ = 2.3, *p* < 0.05]: participants were less aware for Stroop errors when they were stimulated on prefrontal sites than on Vertex (Figure 4B).

The second 3 × 3 ANOVA on Repeat errors did not reveal any effect or interaction (lowest *p*-value = 0.5).

#### Error awareness RTs

The analyses did not show any effect of TMS on *error awareness RTs* (lowest *p*-value = 0.1).

#### Mean RTs

The analysis of *mean RTs* revealed a significant main effect of “kind of response,” [*F*_(1, 19)_ = 38.6, *p* < 0.001, $\eta_{\text{p}}^{2}$ = 0.7]. Error RTs were faster than Go RTs, as in Experiment 1. No main effect or interaction with the factor “site of stimulation” was found (lowest *p*-value = 0.8).

#### Mean accuracy

Finally, similarly to Experiment 1, no significant effect of TMS was found on *mean accuracy* (lowest *p*-value = 0.3).

### Discussion

The results of Experiment 1 failed to confirm our hypothesis about an implication of the right DLPFC on error awareness. The reason for this null finding can encounter several explanations. For example, a simple reason could depend on the inadequacy of the target area, namely the right DLPFC. However, Harty et al. (2014) had strongly demonstrated in two experiments the involvement of the right DLPFC in error awareness and this evidence encouraged us to search a different explanation for the null findings revealed in Experiment 1. Interestingly, the comparison between Experiment 1 and Experiment 2 showed that in Experiment 1 (regardless of the site of stimulation) TMS induced a reduction of the time needed to signal an error. This aspect could be ascribable to an increase of the arousal in participants that were stimulated. In fact, as confirmed in previous studies (Terao et al., 1997; Dräger et al., 2004), TMS can enhance the arousal level, inducing non-specific effects on performance of a task and these effects would not be related to the site of stimulation.

In Experiment 1, it is reasonable to assume that the paired-pulse TMS paradigm may somehow have kept the participants' arousal high during the task because a paired-pulse constantly delivered on the scalp is probably an annoying (or activating) situation. With the aim of control this potential non-specific effect of TMS, in Experiment 3, a single-pulse TMS paradigm was adopted. In Experiment 3, after a response, participants received only a single-pulse TMS instead of two, as in Experiment 1. We hypothesized that the single-pulse TMS paradigm could be a less arousal-inducing paradigm than the paired-pulse.

Although the results were not generalized on error awareness, but only for Stroop awareness, Experiment 3 revealed a potential implication of the DLPFC in error awareness, without evidence for lateralization. These results were partially in contrast with a previous study where the tDCS revealed a selective role of the right DLPFC on error awareness. However, considering the differences between TMS and tDCS, in terms of spatial resolution (Priori et al., 2009), physical and physiological effects (Miniussi et al., 2013), and other distinctions present in the two studies, such as the age of participants (young adults in our study and older adults in Harty's study), this discrepancy in findings may appear less surprising.

Several limitations of the study should be considered when interpreting the present findings. First of all, we must acknowledge that other possible explanations may apply to the null result found in Experiment 1, besides from the non-specific increase of the arousal we suggested. For example, the decision to use a slightly lower intensity of stimulation in Experiment 1 (95% of participants' motor threshold, instead of 100%) could have affected our results. Second, the coordinates proposed by Cieslik et al. (2013) could not be exactly ascribable to individual brains because those coordinates are related to a cluster analysis. Third, a potential limitation of the study concerns the theoretical comparison between Experiment 1 and 3. In fact, the paired-pulse and the single-pulse on-line TMS paradigm we employed in our study were substantially different and, also in the case we had found similar effects of TMS, it would have been difficult to disambiguate any effect, especially because the paradigms were characterized by different timing. Finally, and more important, in Experiment 3 we did not find a generalized effect of TMS on error awareness, as we only found an effect of TMS on Stroop Awareness. Moreover, the small effect size of this interaction points to an increased risk of Type 1 errors and any speculation should be carefully considered after further replications.

## Conclusion

In the present study we aimed to investigate the causal relationship between the right DLPFC and error awareness, as well as to shed light on the timing of error awareness.

This study adds another piece in the puzzle of awareness of cognitive functioning and provides insights into a growing community of researchers that use TMS in cognitive neuroscience and clinical fields. Even if null findings are not easily construable and meaningful, some authors encourage to report and productively interpret null results (Munafò and Neill, 2016) so that they can methodologically guide the design of future TMS research (de Graaf and Sack, 2011).

## Author contributions

FM conception, and design of the study; acquisition, analysis, and interpretation of data; drafting the study; AV, ED, LS, and DM analysis, and interpretation of data; drafting the study and revising it critically; LS technical support for the construction of the computerized task used in the study; DM final approval of the manuscript before the submission.

### Conflict of interest statement

The authors declare that the research was conducted in the absence of any commercial or financial relationships that could be construed as a potential conflict of interest.
